# Naftalan

**Published:** 1899-01

**Authors:** 

**Affiliations:** St. Michael’s Hospital, Tiflis


### Abstracts and Translations.

### NAFTALAN.[1]

BY DR. ROSENBAUM, OF ST. MICHAEL'S HOSPITAL, TIFLIS.

THE medical ointment naftalan is procured from a special and peculiar crude naphtha, differing considerably from any other raw naphtha in its physical and chemical properties. In spite of a high specific gravity of 0.960, this naphtha does not contain any resinous products, dissolves completely in ether, and burns with an agreeable aromatic smell, without leaving any residue. The flash-point of this crude naphtha is beyond 140°, and the point of freezing is 20° C. In the fractional distillation of this naphtha it does not give off light oils, such as benzine, kerosene (petroleum), etc., but immediately as first product (destillate) a heavy oil with a specific gravity of 0.890 is passed over. It can be distilled to dryness without noticing a trace of paraffin.

The place where this peculiar naphtha is found is situated in the Caucasus, at the foot of the Armenic Highlands, and has the Tartaric name, "naftalan." This place is also frequently called the "Holy Bath," because during the three hot months, since time

[1] A paper read at the Imperial Caucasian Medical Society.

immemorial, five to six hundred invalids come yearly, even from far distances, to bathe here in naphtha, seeking cure for all sorts of affections, more especially of diseases of the skin, wounds, rheumatism, gout, etc. The street hawkers of Persia and Asia Minor have always sold the naphtha as a remedy for men and animals.

Naftalan is an extract of this special crude naphtha, and contains the undoubted healing properties of the same in concentration.

This ointment and the oil used with it are prepared without employing any acid or free alkalies, and without admixing any animal or vegetable fat.

The ointment is of stiff consistency, but can nevertheless be easily smeared; it is absolutely neutral, almost odorless, and does not undergo any change, even if kept for years.

The high melting-point of this ointment, which is 60° to 70° C., is of special importance, as it will therefore not liquefy at any body temperature; it covers securely and well, and retains its stiff consistency even in the highest summer temperature. The ointment has, when viewed in reflected light, a dark color; and in a transmitted light a darkish yellow, clear and shiny appearance, and does not leave any stains on the linen after washing.

Naftalan does not mix with water and glycerin, but mixes easily with fat, and is soluble in ether and chloroform. As it never decomposes or splits up, it is excellently suited as a basis for ointments, more especially as it easily mixes with other ingredients, and, contrary to vaseline, is easily and quickly absorbed by the skin.

Naftalan shows strong antiseptic action, in so far that bacteria and their spores cannot exist in it. These are, in short, the so far known properties of the remedy in question. I suppose, however, that still further, up till now unknown, factors will come into consideration, explaining the intense, and in some cases unique, curing effect of this ointment.

Naftalan has been used extensively in St. Michael's Hospital, Tiflis, during the months of March, April, and May, with the following results:

(1) The remedy proved in all cases to be completely harmless, as I never noticed any damaging consequences or by-effects.

(2) It possesses wonderful soothing properties upon burns of the first and second grade, thereby preventing inflammation, and excels, therefore, in this respect all hitherto known remedies.

(3) It acted excellently in various diseases of the skin, especially in acute and chronic eczema, pityriasis, seborrhœa capillitii, and psoriasis, in which cases the remedy produced a marvellous healing effect, even in cases where all other remedies recommended by science had failed.

(4) It favorably influenced the progress of the disease in erysipelas of the face, checking the progressing inflammatory process and reducing the temperature to normal on the second and third days, the patient thereby feeling very well.

(5) It develops an antiseptic, anti-inflammatory action, and accelerates the healing process in inflamed wounds and abscesses.

(6) It is soothing, is readily absorbed, and heals quickly in cases of bruising and sprains, even of long standing.

(7) In rheumatic and gouty pains the patients experience quick relief of the severe pains, and if its use is continued a complete cure is effected.

(8) It has reducing and soothing effect in cases of epididymitis, buboes, and inflamed lymph-glands.

(9) Lastly, naftalan was extensively employed as a basis in the preparation of our mercurial ointment (2 : 1), whereby it was found that the mercury could be more readily incorporated with naftalan than with fat and lanolin. The mercurial ointment prepared with naftalan was readily absorbed by the skin. It is sufficient to smear it on the parts with slight pressure; strong pressure is apt to cause furunculosis, which may be explained by the fact of the sebaceous glands becoming blocked by the mercury. The symptoms quickly disappeared on treatment with the ointment prepared in this way.

The best method to use naftalan is to spread a layer as thick as the back of a knife on linen, and cover the diseased part with it; or the ointment may be placed directly on the diseased part, and then cover with linen or cotton wool. Seeing that the ointment is quickly absorbed by the skin, it is recommended to use it twice a day.—*The Therapist.*

